# Complete mitochondrial genome of *Acheilognathus hypselonotus* Bleekers (Cypriniformes: Cyprinidae) in China’s Dianshan Lake

**DOI:** 10.1080/23802359.2021.1997109

**Published:** 2021-11-10

**Authors:** Lin Zhu, Xuan Che, Xingguo Liu, Xiaodong Wang, Jie Wang, Guofeng Cheng, Xiaolong Chen, Xin Chen

**Affiliations:** a Fishery Machinery and Instrument Research Institute, Chinese Academy of Fishery Sciences, Shanghai, China; b Key Laboratory of Fisher Equipment and Engineering, Ministry of Agriculture, Shanghai, China; c Shanghai Ocean University, Shanghai, China

**Keywords:** Mitochondrial genome, *Acheilognathus hypselonotus*, phylogenetic analysis

## Abstract

The complete mitochondrial genome sequence of *Acheilognathus hypselonotus* is firstly described in this article. The total length of the mitogenome is 16,706 bp. It contains 13 protein-coding genes, 22 tRNA genes and 2 ribosomal RNA genes. The overall base composition of the H-strand is 29.03% A, 25.6% C, 28.13% T, and 17.25% G, with an A＋T bias of 57.16%. The phylogenetic analysis result showed that the *A. hypselonotus*, *A. typus* and *A. macropterus* were a close relationship.

*Acheilognathus hypselonotus* belongs to genus *Acheilognathus* in the family Cyprinidae of Cypriniformes, mainly distributed in the Qiantang River system in China. The body is high lateral flat and oval with a small and pointed head. In the upper part of its body, the posterior margin of each scale is grayish black.

The complete mitochondrial genome of *A. hypselonotus* first determined in this paper was expected to provide help on population genetics of *A. hypselonotus* and further molecular phylogenetic studies. The sample of *A. hypselonotus* in this article was collected from the Huangpu River system in China (121°0′46″E,31°8′35″N). And the specimen was deposited at the Key Laboratory of Fisher Equipment and Engineering, Ministry of Agriculture (contact person Lin Zhu, zhulin@fmiri.ac.cn), under the voucher number Ah20190702023. DNA libraries were prepared using Nextera DNA Flex Library Prep (Illumina, San Diego, CA, USA) and paired-end (150 bp each) sequencing on an Illumina HiSeq X Ten platform. Subsequently, the clean paired-end reads were assembled by the soft ARC (v1.1.4-beta).

The whole length of *A. hypselonotus* mitogenome was 16,706 bp. The nucleotide composition of the heavy strand was 29.03% A, 25.6% C, 28.13% T, and 17.25% G, with an A＋T bias of 57.16%. It contains 13 protein-coding genes, 22 tRNAs and 2 rRNAs. Most genes were located on the heavy strand, but *ND6* and 8 tRNA genes (*tRNA^Gln^*, *RNA^Ala^*, *tRNA^Asn^*, *tRNA^Cys^*, *tRNA^Tyr^*, *tRNA^Ser^*, *tRNA^Glu^*, *tRNA^Pro^*) were encoded on the light strand. Most protein-coding genes initiated with ATG except for COI starting with GTG. It is also important to note that the majority of protein-coding genes is inferred to terminate with an incomplete stop codon T or TA- (ND2, COII, ATPase 6, COIII, ND3, ND4 and Cyt b), five protein-coding genes share the typical termination codon TAA (ND1, COI, ATPase 8, ND4L and ND6); ND5 uses TAG as a stop codon. The length of 12S (located between *tRNA^Phe^* and *tRNA^Val^*) and 16S (located between *tRNA^Val^* and *tRNA^Leu^*) rRNA genes were 957 bp and 1643 bp, respectively.

To investigate the phylogenetic relationship, we downloaded the mitochondrial genome sequences of 16 currently available Cyprinidae species. The concatenated sequences of 13 protein-coding genes, 2 rRNAs genes and 22 tRNAs genes were aligned with the ClustalW program (Larkin et al. 2007). Using the Maximum Likelihood (ML) method (Stamatakis 2006), the phylogenetic tree was constructed (Figure 1) by MEGA6 (Tamura et al. 2013). The best-fitting model (GTR + I + G) was obtained as the optimization model by jModelTest (Posada 2008). The result indicates that the *A. hypselonotus*, *A. typus* and *A. macropterus* were a close relationship (Figure 1).

**Figure 1.**
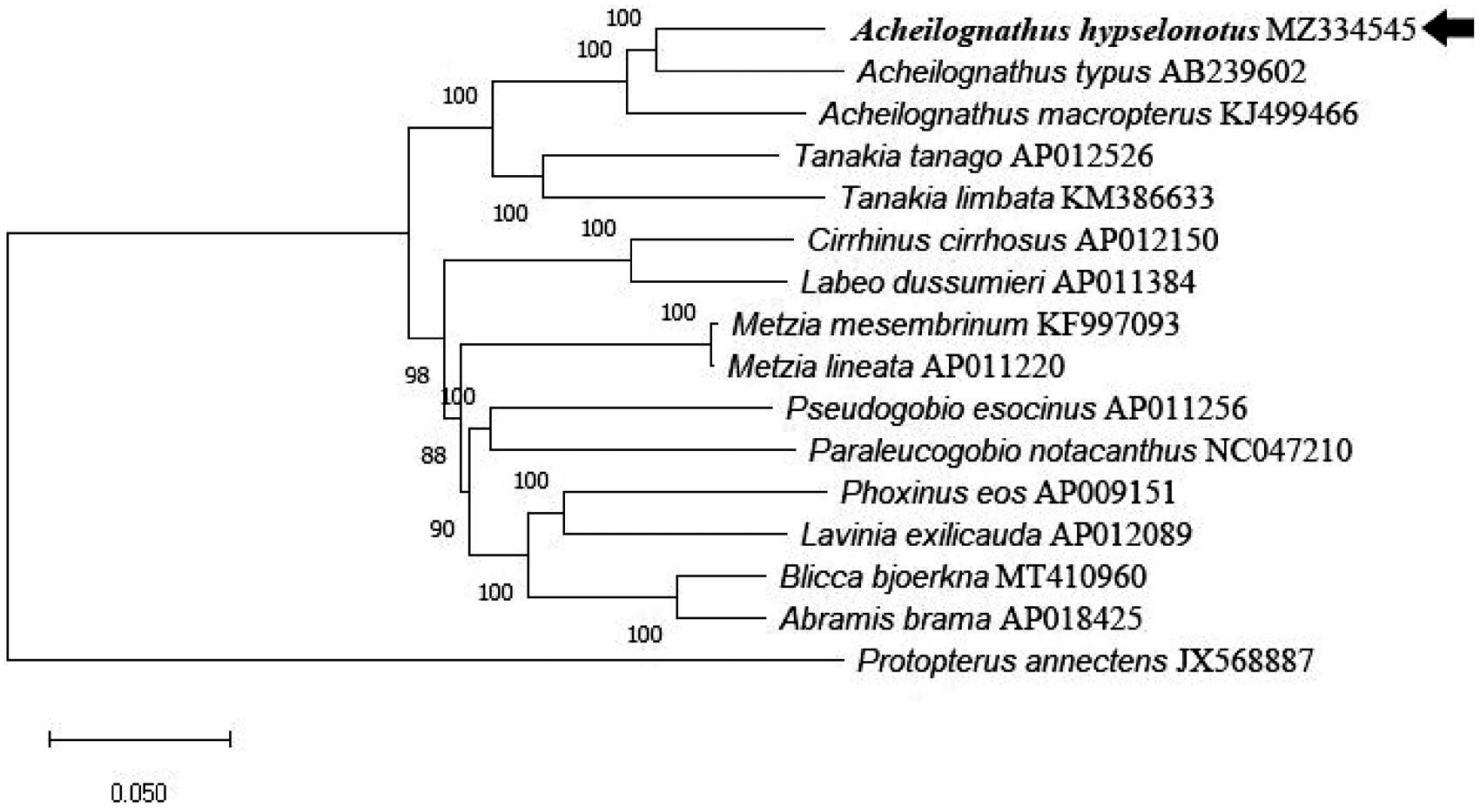
The phylogenetic tree based on the 13 protein-coding genes, 2 rRNAs genes and 22 tRNAs genes of sixteen species. The bootstrap (1000 replicates test) supports for maximum likelihood (ML) method was indicated at each branch. *A. hypselonotus* was marked with a black arrow.

## Data Availability

The genome sequence data that support the findings of this study was openly available on the NCBI website with the accession number MZ334545 (https://www.ncbi.nlm.nih.gov/nuccore/MZ334545). The associated Bioproject, SRA, and Biosample numbers are PRJNA767253, SRR16219791 and SAMN21895032, respectively.
